# Functional Roles of Src Kinase Activity in Oocyte Maturation and Artificial Egg Activation in *Xenopus laevis*

**DOI:** 10.3390/cells15030305

**Published:** 2026-02-06

**Authors:** Ken-ichi Sato, Alexander A. Tokmakov

**Affiliations:** 1 Laboratory of Oocyte Biology, Faculty of Life Sciences, Kyoto Sangyo University, Kamigamo-motoyama, Kita-ku, Kyoto 603-8555, Japan; 2 Faculty of Biology-Oriented Science and Technology, KinDai University, 930 Nishimitani, Kinokawa City 649-6493, Japan; tokmak@waka.kindai.ac.jp

**Keywords:** Src family kinases, *Xenopus laevis* oocyte, oocyte maturation, egg activation, MAPK signaling

## Abstract

**Highlights:**

**What are the main findings?**

- Src kinase activity positively modulates progesterone-induced meiotic maturation in Xenopus laevis oocytes by accelerating MAPK activation and GVBD.
- Src activity is specifically required for membrane-associated egg activation pathways (H_2_O_2_- and Cathepsin B-induced), but not for direct Ca^2+^-mediated activation.

**What are the implications of the main findings?**

- Src functions as a context-dependent signaling amplifier that links membrane-proximal cues to intracellular kinase cascades during oocyte maturation and egg activation.
- These findings clarify how Src selectively integrates maturation and fertilization-like signals, providing a framework for understanding conserved reproductive signaling mechanisms.

**Abstract:**

Src family tyrosine kinases regulate oocyte maturation and fertilization in many species, yet their physiological roles in *Xenopus laevis* (*X. laevis*) remain incompletely defined. Here, we generated three *X. laevis* Src (xSrc) constructs with defined point mutations allowing for selective immunochemical detection and controlled modulation of kinase activity: wild type (xSrcWT, Arg121His), constitutively active (xSrcKA, Arg121His/Tyr526Phe), and kinase-negative (xSrcKN, Arg121His/Lys294Met). Capped mRNAs were microinjected into immature oocytes, and effects on meiotic maturation and egg activation were analyzed. All constructs produced detectable Src protein within 4–5 h after injection without inducing progesterone-independent maturation. Following progesterone treatment, MAP kinase phosphorylation, CDK1 activation, and germinal vesicle breakdown (GVBD) occurred normally in all groups, although xSrcKA-expressing oocytes showed a modest but reproducible acceleration of MAPK activation and GVBD. Global tyrosine phosphorylation analysis revealed increased phosphorylation of several proteins, including a prominent ~50 kDa substrate, specifically in xSrcKA oocytes. After maturation, oocytes were subjected to artificial activation. xSrcKN-expressing oocytes responded normally to Ca^2+^ ionophore (A23187), indicating that Src activity is not required for direct Ca^2+^-mediated activation. In contrast, xSrcKN oocytes exhibited markedly reduced activation in response to hydrogen peroxide or Cathepsin B, which stimulate membrane-associated signaling pathways. These findings demonstrate that Src kinase activity is required for membrane signal-mediated egg activation but is dispensable for activation driven by direct intracellular Ca^2+^ elevation. Collectively, our results identify Src kinase as a positive regulator of progesterone-induced meiotic maturation and a critical mediator of specific fertilization-like activation pathways in *X. laevis*.

## 1. Introduction

Oocyte maturation and egg activation are fundamental transitions in animal reproduction, enabling the progression from prophase I-arrested oocytes to fertilization-competent eggs in a wide range of species, including amphibians and mammals [1,2,3,4,5,6,7,8]. In *X. laevis*, progesterone-induced oocyte maturation is driven by the coordinated activation of maturation-promoting factor (MPF; Cdc2/Cyclin B) and the mitogen-activated protein kinase (MAPK) cascade, culminating in germinal vesicle breakdown (GVBD) and the acquisition of fertilization competence [9,10,11,12,13,14,15,16]. Following maturation, egg activation—triggered either by sperm entry or by artificial stimuli—induces a characteristic rise in intracellular Ca^2+^, cortical contraction, and rapid inactivation of MAPK, thereby promoting exit from meiotic arrest and entry into embryonic mitotic cell cycles [2,3,17]. Although the core components of these signaling pathways have been extensively characterized, the molecular mechanisms by which membrane-associated signals are coupled to intracellular kinase cascades during the transition from oocyte maturation to egg activation remain incompletely understood [18,19,20,21].

Src family kinases (SFKs) have long been implicated in the regulation of oocyte maturation and fertilization in a variety of organisms, including *X. laevis*, sea urchin, starfish, and mammals [22,23,24]. In *X. laevis*, two Src isoforms, xSrc1 and xSrc2, are expressed in oocytes [25,26,27], and early biochemical and physiological studies suggested that Src activation is closely associated with fertilization-induced Ca^2+^ release and egg activation [28,29,30]. These observations positioned Src as a potential key regulator linking membrane-associated fertilization signals to intracellular signaling cascades. Despite these advances, the precise physiological contribution of Src kinase activity to progesterone-induced oocyte maturation, as well as its requirement in distinct egg activation pathways, has not been fully elucidated. Many previous studies relied primarily on pharmacological inhibitors or indirect assays of Src activation, approaches that are limited by off-target effects and do not allow for the controlled manipulation of Src activity levels. Consequently, the biological consequences of defined increases or decreases in Src kinase activity in intact oocytes have remained unresolved. To directly address these issues, we generated three recombinant *X. laevis* Src constructs based on the *xSrc2* gene: wild-type xSrc (xSrcWT), a constitutively active mutant (xSrcKA, Tyr526Phe), and a kinase-negative mutant (xSrcKN, Lys294Met). All constructs additionally carried an Arg121His substitution within the SH3 domain, which enables selective immunochemical detection of the recombinant proteins using the monoclonal antibody mAb327 [31] without interference from endogenous xSrc1/2 [25,32]. Capped mRNAs encoding these constructs were microinjected into stage VI oocytes, allowing for the controlled and experimentally defined modulation of Src kinase activity in a physiologically relevant cellular context.

In this study, we address three central questions. First, does forced expression of wild-type, constitutively active, or kinase-inactive Src alter progesterone-induced meiotic maturation in *X. laevis* oocytes? Second, do differences in Src kinase activity influence downstream signaling events, such as MAPK activation and global protein tyrosine phosphorylation? Third, is Src kinase activity required for specific classes of egg activation signals, and if so, which activation pathways depend on it? By systematically comparing these Src variants under identical physiological conditions, we provide new insights into how Src contributes to oocyte maturation, amplification of intracellular signaling, and egg activation. Our findings resolve several long-standing ambiguities regarding Src function in *X. laevis* oocytes and reveal a previously underappreciated selectivity in Src-dependent egg activation pathways.

## 2. Materials and Methods

### 2.1. Animals and Reagents

Adult female *X. laevis* frogs were purchased from Shimizu Laboratory Supplies (Kyoto, Japan) and maintained in dechlorinated water at 18 °C under a 12 h light/12 h dark cycle. All animal procedures were conducted in accordance with institutional guidelines for animal care and were approved by the local Animal Experiment Committee. A rabbit polyclonal anti-xSrc antibody was generated against a synthetic peptide corresponding to amino acid residues 410–428 of chicken c-Src, as described previously [28]. Polyclonal antibodies against MAP kinase (MAPK) and phospho-MAPK were obtained from Cell Signaling Technology (Beverly, MA, USA). The monoclonal anti-Src antibody (clone 327) was purchased from Oncogene Research Products (Boston, MA, USA). A phosphotyrosine-specific monoclonal antibody (PY99) was obtained from Santa Cruz Biotechnology (Dallas, TX, USA), and an anti-phospho-Cdc2 antibody was purchased from Calbiochem (San Diego, CA, USA). All anti-Src antibodies recognize both *X. laevis* Src1 and Src2 proteins, which share identical amino acid sequences within the relevant epitope regions. Secondary antibodies included rabbit polyclonal anti-mouse IgG (Cappel, Aurora, OH, USA) and alkaline phosphatase (AP)-conjugated goat anti-rabbit IgG (Santa Cruz Biotechnology, Santa Cruz, CA, USA). Protein A–Sepharose was obtained from Amersham Pharmacia Biotech (Uppsala, Sweden). For RNA preparation and manipulation, the mMESSAGE mMACHINE RNA transcription kit and SUPERaseIn RNase inhibitor were purchased from Ambion (Austin, TX, USA). Total RNA was purified using the RNeasy RNA purification kit (Qiagen, Valencia, CA, USA), and first-strand cDNA synthesis was performed using the SuperScript Preamplification System (Gibco BRL, Grand Island, MD, USA). PCR reagents, including the ProStar ULTRA HF system and Pfu DNA polymerase, were obtained from Stratagene (La Jolla, CA, USA). SYBR Green and TaqMan Master Mix kits for real-time PCR were purchased from Applied Biosystems (Foster City, CA, USA). Progesterone was obtained from Sigma-Aldrich (St. Louis, MO, USA). Unless otherwise specified, all other chemicals were of analytical grade and purchased from Nacalai Tesque (Kyoto, Japan), Wako Pure Chemical Industries (Osaka, Japan), or Sigma-Aldrich.

### 2.2. cDNA Cloning and Construction of Expression Plasmids

**Figure 1:**
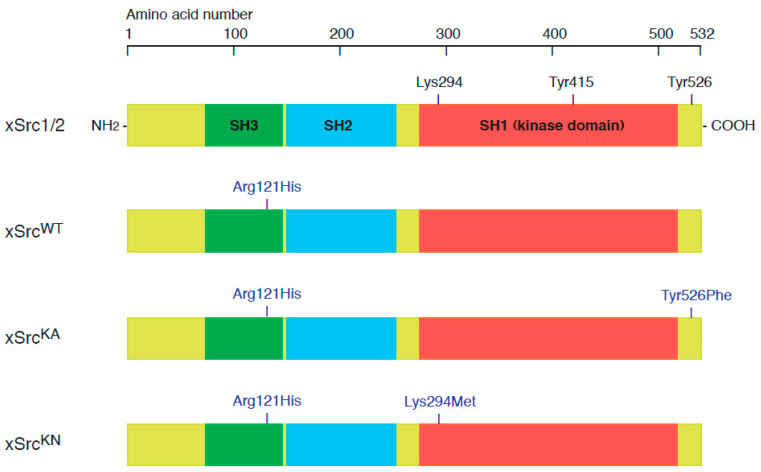
Schematic representation of wild-type and mutant *X. laevis* Src (xSrc) constructs used for expression in oocytes. The full-length xSrc2 protein (accession no. NP_001080738.1) contains three conserved domains: SH3, SH2, and SH1 (kinase domain), with critical regulatory residues Lys294 (ATP-binding site), Tyr415 (autophosphorylation site), and Tyr526 (C-terminal inhibitory phosphorylation site). Three expression constructs were generated: wild type (xSrcWT), constitutively active (xSrcKA, Tyr526Phe), and kinase-negative (xSrcKN, Lys294Met). To enable immunochemical detection distinct from endogenous Src proteins, all constructs included a common Arg121His substitution within the SH3 domain, which renders the recombinant proteins specifically recognizable by the monoclonal antibody mAb327 (anti-human/mouse/chicken Src).

The *X. laevis* orthologue of Src (xSrc) was cloned by reverse transcription–polymerase chain reaction (RT-PCR). Total mRNA was purified from *X. laevis* liver using a QuickPrep Micro mRNA Purification Kit (Amersham Biosciences, Piscataway, NJ, USA). First-strand cDNA was synthesized using the SuperScript First-Strand Synthesis System for RT-PCR (Gibco BRL, Grand Island, MD, USA). The full-length xSrc coding sequence was amplified by PCR using PfuTurbo DNA polymerase (Stratagene, La Jolla, CA, USA) with the following primers: sense primer 5′-AAAAGATCTAGGGCCCATGGGTGCCACTAAAAGCAAGCC-3′ and antisense primer 5′-AAAGGTACCGGGCCCCTAAAGGTTGTCCCAGGCTGGTA-3′. The amplified PCR product was cloned into the pCR-TOPO vector (Invitrogen, Carlsbad, CA, USA) to generate pCR/xSrc, which served as the template for subsequent mutagenesis. Site-directed mutagenesis was performed by oligonucleotide-directed PCR to generate the following xSrc mutant constructs. A kinase-negative mutant (xSrcKN) was generated by substituting lysine 294 in the ATP-binding site with methionine (K294M) using the primers: forward 5′-CCACTCGAGTGGCCATCATGACTCTGAAGC-3′ and reverse 5′-AAAGGTACCGGGCCCCTAAAGGTTGTCCCAGGCTGGTA-3′. A constitutively active mutant (xSrcKA) was generated by replacing tyrosine 526 in the C-terminal regulatory region with phenylalanine (Y526F) using the primers: forward 5′-TTTGGAATTCTCCTGACCGAGCTCACCACC-3′ and reverse 5′-AAAGGTACCGGGCCCCTAAAGGTTGTCCCAGGCTGGAACT-3′. An SH3 domain mutant (xSrcH) was generated by substituting arginine 121 with histidine (R121H) using the primers: forward 5′-CTGGTGGTTGGCACATTCCCTAAGCTCTG-3′ and reverse 5′-CAGAGCTTAGGGAATGTGCCAACCACCAG-3′. To enable selective immunochemical detection of recombinant xSrc proteins, the R121H substitution was introduced into all xSrc constructs. This modification confers immunoreactivity to the monoclonal antibody mAb327, which does not recognize wild-type *X. laevis* Src isoforms due to a single amino acid difference within the epitope region. Our previous study analyzing the epitope region recognized by mAb327 across vertebrate Src family kinases showed that a single amino acid residue within the Src homology 3 (SH3) domain is critical for antibody recognition [25]. In that study, the corresponding position is histidine in human and chicken Src, whereas it is arginine in *X. laevis* Src (and also in human Fyn and Yes), providing a molecular explanation for why mAb327 recognizes human/chicken Src but not native *X. laevis* Src. Consistent with this, the introduction of the Arg121His substitution in our recombinant xSrc constructs confers specific mAb327 reactivity, enabling selective detection and immunoprecipitation of the recombinant xSrc variants (Figure 1). For in vitro transcription and expression in *X. laevis* oocytes, cDNAs encoding wild-type (xSrcWT), kinase-negative (xSrcKN), and constitutively active (xSrcKA) Src were subcloned into the ApaI site of the pBluescript II (SK−) vector (Stratagene) downstream of the T7 promoter.

### 2.3. Preparation of mRNA and Its Expression in X. laevis Oocytes

Capped mRNAs were synthesized in vitro using the mMESSAGE mMACHINE T7 transcription kit (Ambion, Austin, TX, USA) according to the manufacturer’s instructions. Linearized pBluescript II (SK−) plasmids containing the xSrc cDNAs were used as templates for transcription, and 5′ capping was performed using 7-methylguanosine. The synthesized mRNAs were purified using the RNeasy RNA purification kit (Qiagen, Valencia, CA, USA). Polyadenylation at the 3′ end was subsequently carried out using *Escherichia coli* poly(A) polymerase as described previously [33]. After a second purification with the RNeasy kit, mRNAs were dissolved in RNase-free water at a final concentration of 1 mg/mL. RNA integrity and quality were verified by denaturing agarose–formaldehyde gel electrophoresis [34]. Microinjection of synthetic mRNAs into *X. laevis* oocytes was performed essentially as described by Smith et al. [35]. Stage VI defolliculated oocytes were maintained in OR-2 medium containing 82.5 mM NaCl, 2.5 mM KCl, 1 mM CaCl_2_, 1 mM MgCl_2_, 1 mM Na_2_HPO_4_, and 5 mM HEPES–NaOH (pH 7.8). Using a Nanoject microinjector (Drummond, Broomall, PA, USA), 25 nL of mRNA solution (1 ng/nL) or an equivalent volume of RNase-free water (control) was injected into each oocyte. Injected oocytes were incubated at 18 °C for 6–12 h to allow for recombinant protein expression. Following incubation, oocytes were lysed by repeated pipetting in 10 volumes of Buffer A containing 1% Triton X-100 (50 mM Tris-HCl, pH 7.5, 1 mM EDTA, 1 mM EGTA, 10 mM β-mercaptoethanol, 1 mM Na_3_VO_4_, 10 µg/mL leupeptin, and 20 µM *p*-(amidinophenyl)methanesulfonyl fluoride hydrochloride [APMSF]). Lysates were clarified by centrifugation and used immediately for subsequent immunoprecipitation or immunoblotting analyses.

### 2.4. Oocyte Maturation and Artificial Activation Assays

Stage VI defolliculated *X. laevis* oocytes were maintained in OR-2 medium (82.5 mM NaCl, 2.5 mM KCl, 1 mM CaCl_2_, 1 mM MgCl_2_, 1 mM Na_2_HPO_4_, and 5 mM HEPES–NaOH, pH 7.8) at 18 °C. To induce meiotic maturation, progesterone was added to the culture medium at a final concentration of 2 µM, 5 h after microinjection of xSrc mRNAs. Germinal vesicle breakdown (GVBD) was initially monitored by the appearance of a white spot on the animal hemisphere. In addition, GVBD status was assessed morphologically as follows. At designated time points, oocyte samples were fixed in 10% (*v*/*v*) trichloroacetic acid (TCA). Each oocyte was bisected along the animal–vegetal axis using a razor blade, and the two halves were examined under a stereomicroscope with the cut surface facing upward. The presence or absence of the germinal vesicle on the animal hemisphere—retained in immature oocytes—was confirmed and documented by photomicrography. Oocytes in which GVBD was not detected within 8 h after progesterone treatment were classified as immature. For biochemical confirmation of maturation, oocytes were collected at the indicated time points and analyzed for CDK1/Cdc2 dephosphorylation and MAP kinase (MAPK) activation by immunoblotting. To examine egg activation, progesterone-matured oocytes were subjected to artificial activation stimuli. Three activation treatments were employed: (1) calcium ionophore A23187 (10 µM) to directly elevate intracellular Ca^2+^ levels; (2) hydrogen peroxide (H_2_O_2_; 1 mM) to induce tyrosine phosphorylation and secondary Ca^2+^ release; and (3) Cathepsin B (5 µg/mL) to trigger membrane-associated signaling events. Cortical contraction, a Ca^2+^-dependent morphological hallmark of egg activation characterized by constriction of the pigmented cortex at the animal pole, was assessed by stereomicroscopic observation. Oocytes were examined and recorded for up to 15 min following activation treatment to determine the presence or absence of cortical contraction. Activated oocytes were collected at defined intervals after stimulation, homogenized in HB+ buffer (20 mM Tris–HCl, pH 7.5, 1 mM EDTA, 1 mM EGTA, 10 mM β-mercaptoethanol, 1 mM Na_3_VO_4_, 10 µg/mL leupeptin, and 20 µM APMSF), and analyzed by SDS–PAGE followed by immunoblotting. The activation response was evaluated biochemically by the extent of MAP kinase dephosphorylation.

### 2.5. Protein Extraction and Immunoblotting

Protein extraction from *X. laevis* oocytes was performed essentially as described previously [25], with minor modifications. Briefly, oocytes corresponding to a packed volume of 1 mL were homogenized on ice in 3 mL of homogenization buffer containing 20 mM Tris–HCl (pH 7.5), 1 mM EDTA, 1 mM EGTA, 10 mM β-mercaptoethanol, 1 mM sodium orthovanadate, 10 µg/mL leupeptin, and 20 µM *p*-(amidinophenyl)methanesulfonyl fluoride hydrochloride (APMSF), using a Teflon-glass homogenizer. The homogenates were centrifuged at 15,000× *g* for 15 min at 4 °C, and the resulting supernatants were further clarified by ultracentrifugation at 100,000× *g* for 1 h at 4 °C. Clarified extracts were used immediately for immunoprecipitation or immunoblotting analyses. For immunoblotting, 10–20 µL of clarified oocyte homogenates, diluted 1:10 in HB+ buffer, was separated by SDS–PAGE on 10% polyacrylamide gels. Proteins were transferred to polyvinylidene difluoride (PVDF) membranes using a semi-dry blotting apparatus (Bio-Rad, Hercules, CA, USA). Membranes were blocked for 2 h at room temperature in T-TBS buffer (20 mM Tris–HCl, pH 7.5, 150 mM NaCl, and 0.05% Tween 20) supplemented with 3 mg/mL bovine serum albumin. Membranes were incubated with primary antibodies for 2 h at room temperature at the following dilutions: 1:200 for polyclonal anti-xSrc, anti-phospho-MAPK, anti-phospho-Cdc2, and anti-phosphotyrosine (PY99); and 1:100 for monoclonal anti-Src antibody (mAb327: ref. [19]). After extensive washing with T-TBS, membranes were incubated for 1 h with appropriate secondary antibodies: rabbit anti-mouse IgG (1:500; Cappel, Aurora, OH, USA) or alkaline phosphatase-conjugated goat anti-rabbit IgG (1:1000; Santa Cruz Biotechnology, Santa Cruz, CA, USA). Following extensive washing with T-TBS, immunoreactive bands were visualized using a chromogenic substrate solution containing 100 mM Tris–HCl (pH 9.5), 5 mM MgCl_2_, 100 mM NaCl, 50 µg/mL 5-bromo-4-chloro-3-indolyl phosphate p-toluidine salt, and 150 µg/mL nitro blue tetrazolium, and the reaction was terminated once specific bands became clearly visible.

## 3. Results

### 3.1. Generation of Wild-Type and Mutant xSrc Constructs for Expression in X. laevis Oocytes

To investigate the physiological roles of Src during oocyte maturation and egg activation in *X. laevis*, we generated three expression constructs based on the *xSrc2* gene (accession no. NP_001080738.1): wild-type xSrc (xSrcWT), a constitutively active mutant (xSrcKA), and a kinase-negative mutant (xSrcKN). The constitutively active mutant xSrcKA was produced by substituting the C-terminal regulatory tyrosine residue (Tyr526) with phenylalanine, a modification that prevents inhibitory phosphorylation and stabilizes Src in an open, active conformation. In contrast, the kinase-negative mutant xSrcKN was generated by replacing the ATP-binding lysine residue (Lys294) with methionine, resulting in a catalytically inactive Src protein.

Capped mRNAs transcribed from these constructs were microinjected into stage VI *X. laevis* oocytes to achieve transient expression, and the effects of recombinant Src expression on oocyte physiology were subsequently analyzed (see Section 2). To allow for immunochemical discrimination between exogenously expressed and endogenous Src proteins, all constructs (xSrcWT, xSrcKA, and xSrcKN) additionally carried an Arg121His substitution within the SH3 domain. This substitution confers specific recognition by the monoclonal antibody mAb327, which reacts with chicken, mouse, and human Src but does not recognize unmodified *X. laevis* Src proteins, thereby enabling selective detection and immunoprecipitation of the recombinant xSrc variants (Figure 1).

### 3.2. Expression of Recombinant xSrc Proteins and Their Initial Effects on Oocytes

**Figure 2:**
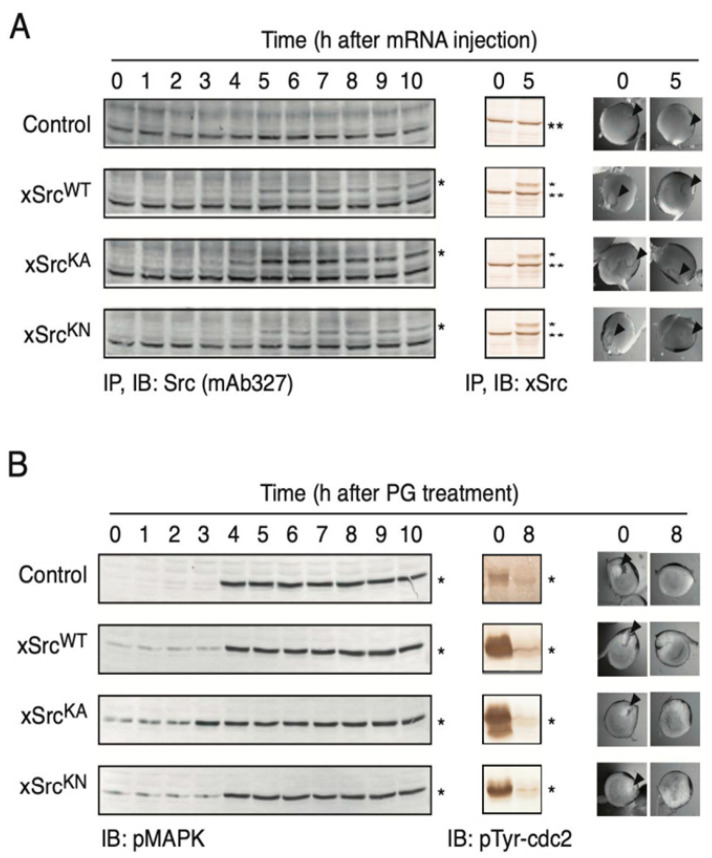
Time-dependent expression of recombinant xSrc proteins in *X. laevis* oocytes following mRNA microinjection, and progesterone-induced MAPK activation, CDK1/Cdc2 dephosphorylation, and GVBD occur normally in oocytes expressing recombinant xSrc proteins. (**A**) Left panels: Recombinant xSrc proteins, as indicated by asterisks (*), were detected by immunoprecipitation (IP) using mAb327 followed by immunoblotting (IB). All constructs became detectable approximately 4–5 h after mRNA injection. Middle panels: Total Src protein levels were examined using an antibody that recognizes both endogenous (**) and recombinant xSrc (*). Recombinant xSrc proteins were expressed at levels comparable to or higher than endogenous Src. Right panels: Representative bright-field images of oocytes at 0 h and 5 h after mRNA injection. No changes in surface pigmentation or germinal vesicle morphology were observed, indicating that forced expression of xSrc constructs did not induce premature maturation or morphological abnormalities. (**B**) Left panels: Immunoblotting for phosphorylated MAP kinase (pMAPK), as indicated by asterisks (*), showing time-dependent activation following exposure to 2 µM progesterone. Middle panels: Immunoblotting for CDK1/Cdc2 phosphorylated on the inhibitory tyrosine residue (pTyr-Cdc2), as indicated by asterisks (*). Dephosphorylation indicates activation of CDK1 and progression toward GVBD. Right panels: Representative images of oocytes at 0 h and 8 h after progesterone treatment. GVBD, visualized as disappearance of the germinal vesicle (arrowheads), occurred in all groups—including xSrcWT-, xSrcKA-, and xSrcKN-expressing oocytes—with kinetics similar to those of control oocytes. Together, these results indicate that forced expression of recombinant xSrc proteins, regardless of kinase activity, does not interfere with the hormonal maturation program of immature oocytes.

To assess the effects of forced expression of xSrc proteins in *X. laevis* oocytes, we first examined the time course of recombinant protein expression as well as changes in oocyte surface morphology and germinal vesicle integrity. As shown in Figure 2A (left panel), immunoprecipitation followed by immunoblotting with the monoclonal antibody mAb327 demonstrated that all three recombinant proteins—xSrcWT, xSrcKA, and xSrcKN—became detectable approximately 4–5 h after microinjection of the corresponding mRNAs.

Using an immunoblotting approach capable of detecting both endogenous and recombinant Src proteins, we further confirmed that the expression levels of the recombinant xSrc variants were comparable to, or greater than, those of endogenous xSrc in untreated control oocytes (Figure 2A, middle panel). Throughout the 5 h period following mRNA injection, no changes were observed in oocyte surface pigmentation or in the morphology of the germinal vesicle, irrespective of recombinant xSrc expression. In addition, no evidence of progesterone-independent maturation or spontaneous germinal vesicle breakdown (GVBD) was detected.

These results demonstrate that all three recombinant xSrc proteins are efficiently expressed at physiologically relevant levels without inducing detectable morphological changes or premature meiotic progression. Thus, microinjection of xSrc mRNAs does not itself disrupt the immature state of *X. laevis* oocytes, providing a suitable experimental system for subsequent analyses of Src function during oocyte maturation and egg activation.

### 3.3. Progesterone-Induced Meiotic Maturation Proceeds Normally in Oocytes Expressing Wild-Type or Mutant xSrc Proteins

To determine whether forced expression of xSrc proteins affects progesterone-dependent oocyte maturation, we examined the response of oocytes expressing xSrcWT, xSrcKA, or xSrcKN to hormonal stimulation. Five hours after mRNA microinjection, oocytes were transferred to a medium containing 2 µM progesterone, and changes in surface morphology were monitored for up to 10 h. In parallel, defined numbers of oocytes were sampled at hourly intervals for biochemical analyses (see Materials and Methods). In addition to assessing germinal vesicle breakdown (GVBD) as a morphological hallmark of meiotic maturation, activation of MAP kinase was evaluated by phosphorylation of its activation-loop threonine and tyrosine residues, and activation of CDK1/Cdc2 was assessed by dephosphorylation of its inhibitory tyrosine residue.

As shown in Figure 2B, oocytes expressing xSrcWT, xSrcKA, or xSrcKN all underwent progesterone-dependent GVBD with kinetics comparable to those observed in control oocytes lacking recombinant Src expression (right panels). Consistent with this morphological progression, MAPK phosphorylation increased progressively following progesterone treatment in all groups (left panels), while CDK1/Cdc2 displayed time-dependent tyrosine dephosphorylation indicative of activation (middle panels).

Together, these results show that forced expression of wild-type, constitutively active, or kinase-negative xSrc does not impair the ability of *X. laevis* oocytes to undergo progesterone-induced meiotic maturation. Thus, neither increased Src signaling nor loss of Src kinase activity disrupts the normal hormonal responsiveness of immature oocytes.

### 3.4. Enhanced Progesterone Responsiveness and Increased Tyrosine Phosphorylation in Oocytes Expressing Constitutively Active xSrc

**Figure 3:**
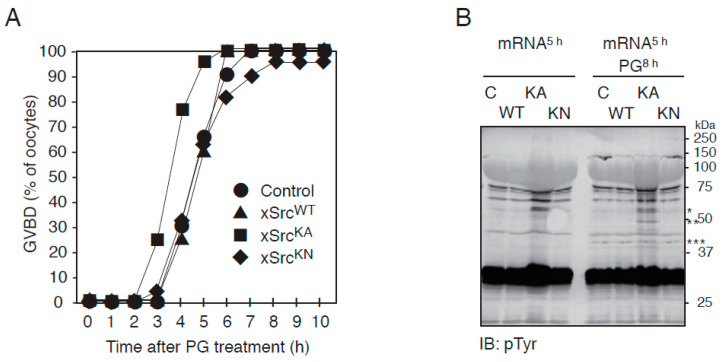
Acceleration of progesterone-induced GVBD and increased tyrosine phosphorylation in oocytes expressing constitutively active xSrc. (**A**) Time course of GVBD in control oocytes and those expressing xSrcWT, xSrcKA, or xSrcKN. Oocytes expressing the constitutively active mutant xSrcKA displayed a modest but reproducible acceleration in GVBD onset compared with other groups. The percentage of GVBD was assessed at 1 h intervals following progesterone treatment. (**B**) Anti-phosphotyrosine immunoblot showing progesterone-induced tyrosine phosphorylation of multiple proteins. Oocytes expressing xSrcKA exhibited increased phosphorylation of several proteins relative to controls and other Src constructs. A strongly phosphorylated ∼50 kDa protein (**), distinct from recombinant xSrc itself (∼60 kDa; *) and MAPK (∼42 kDa; ***), was specifically enhanced in xSrcKA-expressing oocytes. This suggests the presence of an unidentified substrate whose phosphorylation is stimulated by elevated Src kinase activity.

Although progesterone-induced meiotic maturation proceeded with overall similar kinetics in oocytes expressing the three xSrc variants (Figure 2B), a more detailed time-course analysis revealed a modest but reproducible acceleration of the maturation response in oocytes expressing the constitutively active mutant xSrcKA. In three independent experiments using oocytes from three different female frogs (15 oocytes per frog, total n = 45 per condition), quantification of germinal vesicle breakdown (GVBD) at one-hour intervals showed that xSrcKA-expressing oocytes initiated GVBD earlier than control oocytes or those expressing xSrcWT or xSrcKN (Figure 3A). This accelerated morphological response coincided with an earlier increase in MAPK phosphorylation in xSrcKA-expressing oocytes (Figure 2B, left panels).

To further characterize biochemical changes associated with elevated Src activity, we examined global protein tyrosine phosphorylation in oocytes expressing each xSrc construct. Immunoblot analysis using an anti-phosphotyrosine antibody revealed that progesterone treatment led to increased tyrosine phosphorylation of multiple proteins in xSrcKA-expressing oocytes compared with control, xSrcWT-, or xSrcKN-expressing oocytes (Figure 3B). Among these, a prominent phosphoprotein with an apparent molecular mass of approximately 50 kDa (** in Figure 3B) displayed enhanced phosphorylation specifically in xSrcKA-expressing oocytes. This band was distinct from the recombinant xSrc protein itself (approximately 60 kDa; * in Figure 3B) and from MAPK (approximately 42 kDa; *** in Figure 3B).

Together, these results show that constitutive activation of Src is associated with an accelerated progesterone response and increased tyrosine phosphorylation of a subset of proteins during meiotic maturation.

### 3.5. Src Kinase Activity Is Required for H_2_O_2_- and Cathepsin B-Induced Egg Activation but Not for Ca^2+^-Ionophore-Triggered Activation

We next examined whether forced expression of the xSrc constructs affected the ability of progesterone-matured oocytes to undergo egg activation. Five hours after mRNA injection, oocytes were treated with progesterone and incubated for an additional 8 h to allow for full maturation. Matured oocytes were then subjected to three distinct activation stimuli: (1) 2 μM calcium ionophore A23187 for 40 min, (2) 10 mM hydrogen peroxide (H_2_O_2_) for 20 min followed by a 20 min wash, and (3) 5 μg/mL Cathepsin B for 40 min (see Materials and Methods). Egg activation was assessed by monitoring cortical contraction and MAP kinase (MAPK) dephosphorylation.

**Figure 4:**
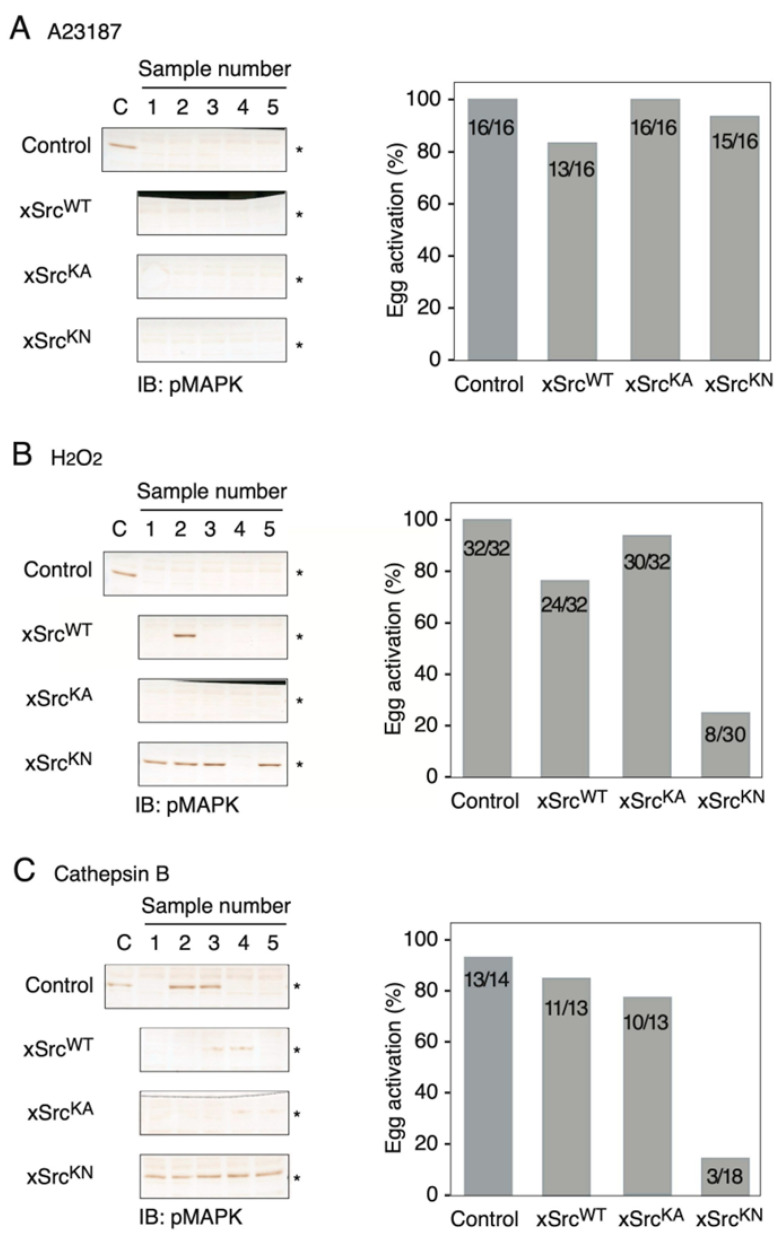
Differential requirements for Src kinase activity during egg activation induced by A23187, H_2_O_2_, or Cathepsin B. Matured oocytes (mRNA injection for 5 h + progesterone treatment for 8 h) were subjected to the indicated activation stimuli. (**A**) A23187-induced activation. Left: MAPK dephosphorylation assessed by anti-pMAPK immunoblotting. Right: Percentage of oocytes undergoing cortical contraction. All three xSrc-expressing groups activated efficiently, similar to controls, indicating that Src kinase activity is not required for Ca^2^+-ionophore-induced activation. (**B**) H_2_O_2_-induced activation. Left: MAPK dephosphorylation profiles. Right: Quantification of cortical contraction. xSrcKN-expressing oocytes showed markedly reduced activation compared to control, xSrcWT, and xSrcKA oocytes. (**C**) Cathepsin B-induced activation. Left: MAPK dephosphorylation following Cathepsin B treatment. Right: Activation rates based on cortical contraction. As with H_2_O_2_, xSrcKN-expressing oocytes displayed a strong reduction in responsiveness, demonstrating the requirement of Src kinase activity in membrane-associated activation signaling. In all panels, asterisks (*) indicate the positions of pMAPK.

Upon treatment with A23187, oocytes expressing xSrcWT, xSrcKA, or xSrcKN all underwent cortical contraction and MAPK dephosphorylation to a similar extent as control oocytes (Figure 4A). Thus, activation induced by direct elevation of intracellular Ca^2+^ occurred independently of Src kinase activity.

In contrast, marked differences were observed when egg activation was triggered by H_2_O_2_ or Cathepsin B. Control oocytes, as well as oocytes expressing xSrcWT or xSrcKA, exhibited robust activation responses characterized by cortical contraction and loss of MAPK phosphorylation. By comparison, oocytes expressing the kinase-negative mutant xSrcKN displayed a substantially reduced activation response under both conditions (Figure 4B,C).

## 4. Discussion

In this study, we demonstrate that regulated Src kinase activity plays a dual role in *X. laevis* oocyte physiology: it modulates the timing and signaling efficiency of progesterone-induced meiotic maturation, and it is required specifically for membrane-associated egg activation pathways, but not for activation driven by direct intracellular Ca^2+^ elevation. These findings clarify the functional significance of Src in both meiotic signaling and egg activation, while also defining clear boundaries for its physiological involvement.

Comparative analysis of the amino acid sequences of *X. laevis* Src1 (NCBI Reference Sequence: NP_001079114.1) and Src2 (NCBI Reference Sequence: NP_001080738.1) reveals an extremely high degree of identity (>98%) across the full-length proteins, with differences limited to a small number of conservative amino acid substitutions and a short insertion [25,26,27]. This high level of sequence conservation indicates that Src1 and Src2 are essentially equivalent in terms of three-dimensional structure and functional domains. Given this structural similarity, forced expression of Src2 is expected to generate a protein capable of functionally competing with, or interfering with, not only endogenous Src2 but also Src1 in *X. laevis* oocytes. From a molecular and structural standpoint, it is therefore reasonable to conclude that overexpressed Src2 can act in a dominant-active or dominant-negative manner with respect to the endogenous Src1/2 pool. This high degree of functional redundancy likely explains why expression of kinase-negative Src2 does not completely abolish maturation-associated signaling, while still revealing pathway-specific requirements for Src kinase activity under defined activation conditions.

First, we show that forced expression of recombinant xSrcWT, xSrcKA, or xSrcKN does not disrupt the immature state of prophase I-arrested oocytes. Neither cortical morphology nor germinal vesicle integrity was altered by Src overexpression, indicating that increased Src abundance alone is not sufficient to perturb the immature state of prophase I-arrested oocytes. Progesterone-induced meiotic maturation proceeded with normal kinetics in oocytes expressing xSrcWT or xSrcKN, consistent with previous observations that Src activity is not strictly required for hormonal maturation in *X. laevis* oocytes. In contrast, oocytes expressing the constitutively active mutant xSrcKA reproducibly exhibited earlier MAPK activation and accelerated GVBD. This phenotype suggests that Src kinase activity acts as a positive modulator of the progesterone signaling network, potentially by facilitating MAPK activation or by sensitizing upstream regulators such as Mos, Raf, or Cdc25 [11,17,32,36]. During *X. laevis* oocyte maturation, Src appears to function as an upstream integrative factor that shapes both the magnitude and the temporal dynamics of downstream signaling. As demonstrated by Tokmakov et al. [32], Src kinase activity is tightly regulated during oocyte maturation through dynamic changes in its phosphorylation state and interactions with regulatory partners. Importantly, this regulated activation of Src does not simply serve as an on–off trigger but rather acts as a critical signaling node that channels and amplifies upstream inputs into the Ras–Raf–MEK–MAPK cascade. In this framework, Src may promote MAPK activation either indirectly—by modulating upstream components such as Mos or Raf—or by facilitating signaling environments that favor sustained MAPK activation. Thus, Src can be viewed as a signaling hub that integrates multiple upstream cues into a coherent output required for meiotic progression, with MAPK representing a principal downstream effector. Consequently, quantitative and temporal control of Src activity is likely a key determinant of MAPK activation dynamics and, ultimately, of successful *X. laevis* oocyte maturation.

In addition, enhanced tyrosine phosphorylation of a ∼50 kDa protein was observed specifically in xSrcKA-expressing oocytes during progesterone-induced maturation. Although the precise identity of this protein remains undetermined, its phosphorylation profile is consistent with that of a Src-responsive substrate and suggests that elevated Src activity amplifies specific signaling cascades associated with meiotic progression. Notably, preliminary immunoprecipitation followed by re-blotting analyses using phosphotyrosine-specific antibodies and additional candidate-specific antibodies suggest that this phosphoprotein may correspond to Shc (Src homology and collagen) protein, a well-known adaptor molecule downstream of Src family kinases [37,38,39,40]. However, as this evidence is not yet definitive, we have refrained from assigning a firm molecular identity in the present study. Definitive identification of this phosphoprotein, ideally by immunoprecipitation followed by mass spectrometry, will be an important subject for future investigations aimed at elucidating the molecular mechanisms by which Src modulates maturation-associated signaling pathways.

Second, while progesterone-induced meiotic maturation was largely independent of Src kinase activity, egg activation displayed a pronounced Src dependence that varied according to the nature of the activating stimulus. Activation induced by the calcium ionophore A23187—which directly elevates intracellular Ca^2+^ and bypasses membrane-associated signaling—was unaffected by expression of any xSrc construct, including the kinase-negative mutant. In contrast, activation induced by hydrogen peroxide (H_2_O_2_) or by Cathepsin B—both of which engage membrane-proximal signaling events—was strongly impaired in oocytes expressing xSrcKN. These observations indicate that Src kinase activity is dispensable for Ca^2+^-ionophore-triggered egg activation but is required for activation pathways that depend on upstream membrane-associated signals [41,42,43,44,45]. Importantly, these artificial activation models are thought to mimic distinct aspects of physiological fertilization signaling. H_2_O_2_ is believed to promote a rapid increase in protein tyrosine phosphorylation—consistent with activation of Src-family kinases—and thereby to initiate downstream PLCγ-dependent Ca^2+^ release. Cathepsin B, in turn, is proposed to mimic fertilization-like membrane proteolysis by partially cleaving an egg surface protein such as uroplakin III, triggering a membrane-initiated Src→PLCγ signaling cascade. Thus, the selective requirement for Src activity in H_2_O_2_- and Cathepsin B-induced activation supports the view that Src is specifically engaged when egg activation is initiated by membrane-proximal oxidative and/or proteolytic cues, rather than by direct cytosolic Ca^2+^ elevation. The preserved responsiveness of xSrcKN-expressing oocytes to A23187 further supports the conclusion that the reduced activation observed with H_2_O_2_ and Cathepsin B reflects pathway-specific requirements rather than a general loss of oocyte competence due to xSrcKN overexpression.

Taken together, our results support a model in which Src functions as a flexible signaling amplifier rather than an obligatory trigger during *X. laevis* oocyte maturation and egg activation. Progesterone-induced maturation can proceed in the absence of Src kinase activity, although elevated Src activity enhances the efficiency and timing of meiotic transitions. In contrast, egg activation requires Src activity only when the initiating stimulus depends on receptor-mediated or proteolytic mechanisms at the plasma membrane, such as those engaged by oxidative treatment or Cathepsin B-dependent cleavage events [43,44]. This dual, context-dependent role of Src highlights the evolutionary advantage of integrating Src-dependent membrane signaling with robust intracellular kinase cascades that govern meiotic resumption and egg activation.

The view of Src as a context-dependent “signaling amplifier” in *X. laevis* oocytes is also consistent with broader work on Src-family kinases across animal species [46,47,48,49,50,51,52,53]. In many externally fertilizing organisms, Src-family kinases have been implicated in linking sperm–egg interactions at the plasma membrane to intracellular signaling events that culminate in Ca^2+^ release and egg activation. Studies in sea urchin and starfish eggs, for example, support roles for SFKs in early fertilization-associated tyrosine phosphorylation and activation of downstream effectors involved in Ca^2+^ mobilization and cell-cycle transitions [46,47,53]. In mammals, SFKs have likewise been connected to signaling events during oocyte maturation and/or fertilization, although the upstream triggers and the relative contribution of individual family members can differ depending on species and experimental context [48,49,50,51,52]. Together, these cross-species observations suggest that a conserved feature of Src-family function in oocytes is the capacity to amplify membrane-initiated signals into robust intracellular kinase and Ca^2+^ responses, while the precise dependence on Src activity may vary with the nature of the initiating cue and the degree of redundancy among paralogous SFKs.

Finally, regarding the fate of progesterone-matured oocytes during prolonged culture, previous studies have shown that unfertilized Xenopus eggs undergo spontaneous apoptotic degeneration accompanied by MAPK inactivation [54,55]. In the present study, we did not include dedicated time-course analyses specifically designed to quantify this late-stage post-maturation degeneration. Nevertheless, in our laboratory observations, matured oocytes across all xSrc expression conditions displayed comparable late-stage morphological deterioration during extended incubation, and caspase activation was detectable under these conditions (unpublished observations). Because these observations were not systematically quantified here, we refrain from drawing definitive conclusions regarding the contribution of Src kinase activity to long-term post-maturation apoptosis. Further targeted studies will be required to elucidate the molecular mechanisms governing unfertilized-egg apoptosis and to assess potential roles of Src, uroplakin III-dependent signaling, and related pathways.

Collectively, this work provides a mechanistic framework for understanding the context-specific contributions of Src to oocyte biology and identifies new avenues for investigating the molecular substrates and pathways that underlie Src-dependent activation signaling. A widely used strategy for elucidating the physiological functions of a given gene product is the application of genetic knockout or loss-of-function approaches [56,57,58,59,60]. However, in the case of Src family kinases (SFKs), interpretation of knockout phenotypes is frequently complicated by the presence of multiple closely related paralogs with partially overlapping biological functions [24,61,62,63,64]. The landmark study by Soriano and colleagues demonstrated that Src-null mice are viable but exhibit severe osteopetrosis, revealing an essential role for Src in osteoclast function while simultaneously indicating that other SFK members compensate for many aspects of Src signaling in most tissues [65]. Similar conclusions have been drawn from double- and triple-knockout studies of SFKs, in which removal of a single isoform often fails to produce pronounced phenotypes due to functional redundancy and compensatory upregulation of remaining family members [66,67]. These observations underscore a fundamental limitation of genetic loss-of-function analyses: the absence of an overt phenotype does not necessarily imply the absence of physiological function, particularly within gene families characterized by high structural and functional similarity. In this context, our experimental strategy—using precisely engineered, epitope-tagged wild-type, constitutively active, and kinase-negative Src mutants expressed in a system that naturally contains multiple SFK paralogs—provides a complementary approach that enables dissection of Src-specific functions without being obscured by endogenous redundancy.

Although this study provides clear evidence for a functional contribution of Src kinase activity to artificial egg activation, we were unable to directly assess its role during fertilization itself. This limitation reflects a well-recognized technical challenge in *X. laevis*: oocytes matured in vitro by progesterone treatment do not readily support normal fertilization or embryogenesis when subjected to standard in vitro fertilization procedures [68,69]. While a specialized protocol developed by Heasman and colleagues enables progesterone-matured oocytes to be rendered fertilizable, its implementation is technically demanding and was not feasible within the scope of the present study. Consequently, our conclusions regarding Src function during egg activation are confined to artificial activation paradigms, and the physiological relevance of Src kinase activity during sperm-triggered egg activation remains an important subject for future investigation.

## 5. Conclusions

In this study, we employed a set of recombinant *X. laevis* Src proteins—wild-type, constitutively active, and kinase-negative—to dissect the functional roles of Src kinase activity during oocyte maturation and egg activation. Our results demonstrate that elevated Src activity enhances the efficiency and timing of progesterone-induced meiotic maturation, whereas loss of Src kinase activity does not impair the maturation program itself. In contrast, Src activity is required for membrane-mediated egg activation triggered by stimuli such as hydrogen peroxide or Cathepsin B, but is dispensable for activation induced by direct intracellular Ca^2+^ elevation. Together, these findings reveal a context-dependent requirement for Src in oocyte signaling, in which Src functions as a positive modulator of meiotic signaling and as a critical mediator of specific membrane-associated activation pathways. This work provides a clearer mechanistic framework for understanding how Src integrates upstream cues during the transition from oocyte maturation to egg activation in *X. laevis*.

## Data Availability

The original contributions presented in this study are included in the article. Further inquiries can be directed to the corresponding author.
